# Comment on “In vivo flow cytometry reveals a circadian rhythm of circulating tumor cells”

**DOI:** 10.1038/s41377-021-00624-4

**Published:** 2021-09-17

**Authors:** Amber L. Williams, Jessica E. Fitzgerald, Fernando Ivich, Eduardo D. Sontag, Mark Niedre

**Affiliations:** 1 Department of Bioengineering, Northeastern University, Boston, MA USA; 2 Department of Electrical and Computer Engineering, Northeastern University, Boston, MA USA; 3 Laboratory of Systems Pharmacology, Harvard Medical School, Boston, MA USA

**Keywords:** Imaging and sensing, Confocal microscopy

Dear Editor,

Circulating tumor cells (CTCs) are instrumental in hematogenous metastasis and are widely studied using liquid biopsy methods. These involve analysis and characterization of CTCs from fractionally small blood samples drawn from patients^1^. Liquid biopsy implicitly assumes that the number and phenotypical distribution of CTCs in small blood samples is representative of the full peripheral blood volume, and furthermore that CTC numbers are approximately constant over the days and hours surrounding the blood draw.

Recently, Zhu^2^ et al. reported in *Light* that this assumption is at times dubious. In particular, they used a fluorescence microscopy method called “in vivo flow cytometry” (IVFC) to show that the number of CTCs in circulation may indeed vary significantly over the short term. In particular, they demonstrated a significant circadian effect in a mouse prostate cancer model, and temporal behavior that is significantly more dynamic than is generally assumed. As a result, enumeration of CTCs from small blood samples could lead to significant under- or over-estimation of CTC numbers.

The authors appear to be unaware of prior work published by our group^3^ and by Juratli et al.^4^, neither of which were cited. Our paper^3^ presented similar in vivo optical measurements (using “diffuse in vivo flow cytometry”), in mouse xenograft models, which was based on long-standing research at our lab at Northeastern University^5^. We explicitly showed significant short-term (minutes, hours, days) fluctuations in tumor cell numbers in circulation, and that CTC detection statistics deviated from Poisson which are widely assumed^6^. We also demonstrated the superiority of multi-sample averaging approaches in accurate quantification of CTC numbers. We did search for circadian variations in CTC numbers in a multiple myeloma mouse xenograft model, but did not observe one as did Zhu et. al. in a prostate cancer model^2^. Likewise, Juratli et al.^4^ used microscopy-IVFC and observed that CTC numbers in mice in general did not correlate with tumor size, and that the rate of CTC detection using their instrument was highly variable over short time periods. They showed that this was true in epithelial and non-epithelial tumor models.

We believe that these works and others reflect important insights into short-term circulation dynamics of CTCs that are currently poorly understood and may ultimately contribute to a more complete understanding of hematogenous metastasis. We are also gratified that multiple teams of investigators could independently confirm similar results in different animal models. However, we do believe that the scientific record should be corrected to reflect prior contributions to the field.
